# Reading and Interpreting the Histone Acylation Code

**DOI:** 10.1016/j.gpb.2016.12.001

**Published:** 2016-12-19

**Authors:** Jelly H.M. Soffers, Xuanying Li, Susan M. Abmayr, Jerry L. Workman

**Affiliations:** 1 Stowers Institute for Medical Research, Kansas City, MO 64111, USA; 2 Department of Anatomy and Cell Biology, University of Kansas Medical Center, Kansas City, KS 66160, USA

Decades of research has explored the epigenetic control of gene expression and the impact of histone post-translational modifications (PTMs), such as acetylation, on chromatin remodeling. Indeed, the writers, readers, and erasers of lysine acetylation are increasingly well understood. Recent studies have added crotonylation, butyrylation, and propionylation to the types of acylations by which histones are modified, and identified the YEATS protein domain as a critical reader of crotonylation. Now, Haitao Li, David Allis, and their colleagues expand the scope of protein domains capable of reading crotonyl-lysine (Kcr) to include double PHD finger (DPF) domains. Importantly, the mechanism through which these domains recognize Kcr is quite distinct from their recognition by the YEATS domain [1]. In this highlight, we discuss recognition of acylated histones by the bromodomain (BRD), the YEATS domain, and PHD fingers. We contrast the structural basis for their recognition of histones modified by acetylation and more recently discovered histone crotonylation [2], [3], [4], [5], [6].

## Roles of histone acylation

Histone acylations are a group of chemical modifications that occur at lysine residues. Lysine acetylation, the addition of –CH_3_CO, represents only one class of possible lysine acylations (KC = OR). Structurally larger acylations include propionylation (three-carbon side chain), butyrylation, and crotonylation (both four-carbon side chains).

Fundamental questions include what drives these different modifications, and what are their biological consequences. The constellation of histone acylations is strongly linked to metabolism. The majority of substrates for histone acylation are acetyl-CoA-linked metabolic intermediates. The activity of acetyl-CoA synthetases increases upon high sirtuin (histone deacetylase; HDAC) activity, which depends on the cellular NAD/NADH ratio [7], [8]. Thus, the metabolic state of the cell is a strong determinant of what acylations are present at what residues in what combination at a given time. Hence, there is the possibility that the pattern of histone acylation may simply reflect the metabolic state of the cell. However, the other possibility is that many histone-acyl PTMs are actively read, and signal for changes in chromatin structure or function. In this light, various acylations could reflect a delicate mechanism that fine-tunes gene expression to metabolic need [9], [10].

Although histone acetylation and crotonylation globally co-localize and mark open chromatin in resting somatic cells, recent evidence suggests that specific acylations serve distinct functions. Some acylation marks are differentially susceptible to histone acetyltransferase (HAT) overexpression. Notably, the Kcr signal is not increased upon overexpression of CBP or p300 [11], two proteins with intrinsic HAT activity. Moreover, histone acylations have different resistance to HDAC activity, which contributes to the formation of subsets of gene loci marked favorably for gene expression [11], [12]. Crotonylation is more resistant to removal, and forms a strong transcription activating mark at testis-specific genes that escape post-meiotic sex inactivation [11], [13]. At the level of individual histone lysine residues, most lysine residues can be modified by several different acylations. However, some sites are mutually exclusive for acetyl-lysine (Kac) or Kcr [14]. Thus, a better understanding of how these various acyl marks translate into changes in chromatin function is necessary.

## Readers of histone acetylation

The reader functions of histone modifying complexes rely on signature domains that specifically recognize and bind histone marks [15]. To date, three domains have been reported to recognize Kac.

BRDs are found in diverse nuclear proteins, including HATs (such as GCN5 and PCAF), ATP-dependent remodeling complexes (such as the SWI/SNF-associated Baz1b and SMARCA2/4), methyltransferases (MLL and ASH1L), and various mediators of transcriptional activity [16]. BRDs form a conserved structural fold of four bundled left-handed helices and two inter-helical loops that constitute an aromatic, hydrophobic pocket [2]. Kac binding relies on conserved amino acid residues, and is supported by water-mediated hydrogen bonds. [2].

A limited number of YEATS domain-containing proteins are associated with HAT complexes, chromatin-remodeling complexes, or transcription-regulating complexes [17]. For example, the YEATS domain of AF9, a component of the human super elongation complex (SEC), binds to acetylated histone H3 with a strong preference for H3K9ac [4], [6], [18]. By adopting an immunoglobulin fold, the AF9 YEATS domain utilizes a serine-lined aromatic ‘‘sandwiching’’ cage for the specific readout of Kac within the RK consensus sequence [4]. This structure ensures an intimate encapsulation of Kac, which is achieved by relayed hydrogen binding, CH-π interactions, and hydrophobic contacts with a high degree of shape complementarity [4].

A large number of PHD-finger containing proteins recognize both modified and unmodified histones, along with non-histone proteins [19]. A small subclass of double PHD fingers is highly specific for histone acetylation marks [19]. Notably, recognition of H3K14ac requires alignment of the histone peptide across a shared surface of the DPF, as has been described for human DPF3b, a component of the BAF chromatin remodeling complex, and the DPF of the HAT MOZ/MORF/MYST3 [3], [20], [21], [22], [23]. At this interface, the N-terminal residues (R2-K4) from H3 form an anti-parallel β-sheet that contributes to the β-sheet of PHD2. Notably, the MOZ DPF induces an α-helical conformation in the H3 tail, which brings a stretch of N-terminal residues close together for PTM sampling [22]. H3K14ac itself interacts with the hydrophobic pocket of PHD1 [3], [20], [21], [22], [23].

## Readers of histone crotonylation

Kcr is not only a fairly large acylation mark, it is also distinct from other acylation marks by the π-electron conjugation of its crotonylamide group. This arrangement gives the PTM an extended ligand conformation that is both rigid and planar in shape. Proper recognition requires non-distortion of this planar orientation [6]. BRDs [9], YEATS domains [6], and the recently-reported DPF domains [1] all have Kcr binding capacity, albeit through different mechanisms and with different affinities.

**Figure 1:**
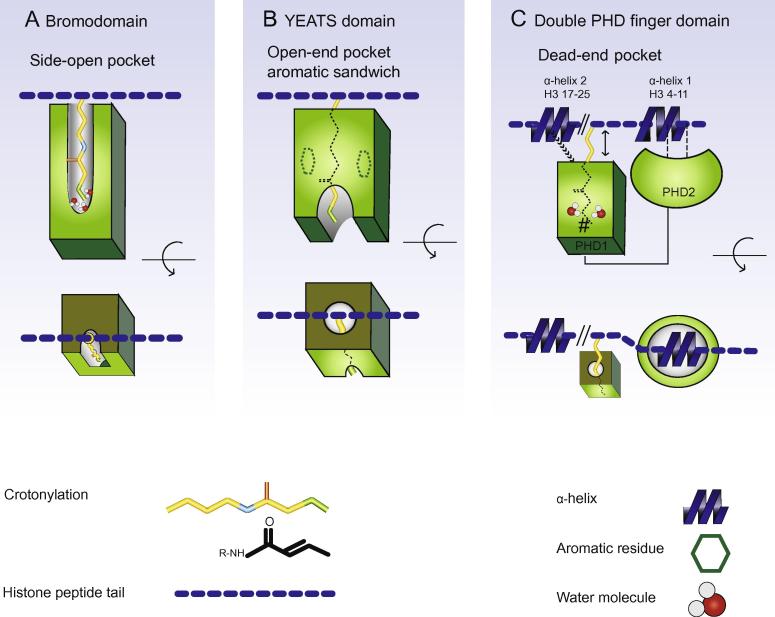
**Schematic overview of the recognition modes of crotonyl-lysine by the bromodomain, YEATS domain, and double PHD finger domainA.** The bromodomain (BDR) adopts a side-open pocket for recognition of histone acylations. Four bundled left-handed helices and two inter-helical loops constitute an aromatic, hydrophobic pocket for binding to a wide range of histone acetylations. However, only the BDR of Taf1(2) accommodates large acylations like Kcr, because it is capable of rearranging the water network deep within the pocket (indicated with water molecules). Other BDRs cannot accommodate Kcr in co-planar fashion, and fail to bind to them with high affinity. **B.** The YEATS domain is an open-end pocket that binds to Kcr with higher affinity than Kac by means of aromatic stacking [6]. **C.** The double PHD fingers of MOZ interact with each other and cooperatively bind to H3K14ac. PHD2 induces an α-helix at H3K4-T11 [22], while H3 and K4 (indicated with dashed lines) interact with the β1 of PHD2. PHD1 induces second α-helix and forms a binding pocket for Kcr, allowing H3L20 and K23 to associate with the so-called I128A and L213A saddle pair of β2 (arrow headed line). DPF-acetyl-lysine recognition depends on a tight fit, brought about by intimate hydrophobic encapsulation (indicated with water molecules) and coordinated hydrogen bonding. This generates a larger pocket and specific interactions that favor Kcr. However, even though residues with small side chains circumvent steric hindrance and allow co-planer insertion of Kcr within the β2 pocket (approximated with the pound sign), the large acylations still stick out of the dead-ended reading pocket (double-arrow headed line). Surprisingly, this does not compromise binding affinity.

The reader pocket of BRDs forms a shaft that is open along the longitudinal axis but has a closed end, a so-called “side open” configuration (Figure1**A**). Low affinity binding to the increasingly longer acylation chains that are added by propionylation, crotonylation, and butyrylation can be accommodated when the extended hydrocarbon chain curls up at the deepest point of insertion. Alternatively, a larger hydrophobic pocket also allows better recognition and binding of these acylations. The gatekeeper residue (flanking the BC-inter-helical loop) determines the size of this pocket. The larger hydrophobic pocket formed with tyrosine gatekeepers (as found in the BRDs of CECR2 and BRD9) accommodates the flexible 4-carbon butyryllysine (Kbu) group. Nevertheless, this pocket still deflects the rigid four-carbon Kcr group from being optimally coplanar, contributing to the low affinity of BRD9 for Kcr [9]. In contrast to the BRDs of CECR2 and BRD9, the second BRD of Taf1, later referred to as Taf1(2), can bind to Kbu and Kcr in a co-planar conformation, since these modifications displace water molecules from their usual positions within the pocket, consistent with a higher-affinity interaction [9].

The YEATS domain, originally identified as a novel family of Kac reader molecules, has now been found to have a broader acyl-binding capacity and a preference for Kcr over Kac [6], [24]. The AF9 YEATS binds to H3K9cr or H3K18cr through an aromatic-sandwiching cage, in which the crotonylamide plane is sandwiched by aromatic residues, π-aromatic stacking, and hydrophobic contacts (Figure1**B**) [6]. In contrast to the BRD reading pocket, the reader pocket of YEATS is elongated and open-ended, increasing its ability to accommodate a large repertoire of acyl-lysine marks with extended acylation chains [6].

Haitao Li, David Allis, and their colleagues [1] now demonstrate that H3K14ac can be recognized and bound by the DPFs in the MOZ/MORF HAT and in the non-catalytic DPF2 subunit of the BAF ATP-dependent nucleosome remodeling complex. Most importantly, these DPFs use a highly-selective pocket to recognize the recently-identified histone Kcr mark, and bind in a manner that is different from that of the YEATS domain. Combining information from crystal structures, isothermal titration calorimetry, and functional assays, this elegant work describes the salient features of DPF structure and interaction with Kcr.

In brief, the tandem PHD fingers of MOZ and DFP2 fold compactly into one unit that surveys histone tails and is able to recognize and bind specific histone acylations. As noted above for the Kac reading mechanism of a single PHD finger, the β1 surface of PHD2 within MOZ recognizes histone H3R2 and H3K4 within the induced α-helix of the N-terminal part of the histone tail. The actual interaction with H3K14ac relies on the β2 pocket of PHD1. This work is the first to demonstrate that PHD1 interacts with a second induced α-helix of H3, which might facilitate interactions with various acylations. The β2 surface of PHD1 forms a closed-end hydrophobic pocket that fits like a glove around the rigid and planar crotonylation side chain (Figure1**C**). This reader pocket is highly size selective. This fit, shown for H3K14cr, is brought about by substituting bulky residues in the β2 pocket with the side-chain-free glycine. This substitution is conserved among the PHD1 domains of human MOZ, MORF, DPF1, DPF2, DPF3, and PHF10, suggesting a common structure that can better accommodate large side chains. Nonetheless, even proteins with structurally-different DPFs exhibit different binding affinities for Kcr. Notably, DPF2 has a higher binding affinity than MOZ, and mutation analysis demonstrates that substitution of critical residues can convert the binding affinity of MOZ to that of DPF2 and *vice versa*. Hydrogen bonds between the crotonylamide group and these critical residues trigger a rotational shift of the K14cr side chain and the rearrangement of three water molecules around the carbonyl group of crotonylamide. Reminiscent of Taf1(2) [9], these changes in the configuration of water molecules create a better fit for Kcr. In addition, the actual number of water molecules can influence which acylations are bound. This ability to accommodate different acyl-lysines has been shown for the MOZ DPF, where more water molecules in the β2 reader pocket facilitate binding of larger modifications. One final unique feature of DPFs is their ability to bind large acylations with moderate to high affinity, even in the absence of deep pockets (Figure1C).

## Conclusion

BRDs, YEATS domains, and DPFs read various histone acyl-lysines with different recognition mechanisms and binding affinities. BRDs adopt a ‘side-open’ pocket that can bind to histone acetyl-lysine with high affinity, but is generally unable to bind to large, rigid residues like crotonylation due to steric hindrance. The YEATS domain uses an ‘end-open’ aromatic sandwich pocket to bind to acylated amino acid residues, which favors Kcr over Kac. Lastly, the DPFs of MOZ and DPF2 employ a ‘dead-end’ hydrophobic pocket for Kcr-specific recognition. Like YEATS domains, DPFs prefer Kcr over Kac, but the basis for this selectivity is different. Specifically, DPFs exploit intimate hydrophobic encapsulation and coordinated hydrogen bonding, generating a larger pocket and specific interactions that favor Kcr.

The cell context-dependent constellation of histone modifications encodes a wealth of information. We are only just beginning to unravel the specific reading mechanisms. The fact that different domains are able to bind to the same histone acylation brings into question what determines which domain will bind under what circumstances. Thus, dissecting recognition mechanisms for histone acylations will broaden our understanding of how histone modifications signal for changes in chromatin accessibility and regulate gene expression.

## Competing interests

The authors have declared no competing interests.
